# Maintaining Tooth Vitality With Super Minimally Invasive Pulp Therapy

**DOI:** 10.7759/cureus.29712

**Published:** 2022-09-28

**Authors:** Yuki Kojima, Ryozo Sendo

**Affiliations:** 1 Anesthesiology, Asahi General Hospital, Asahi, JPN; 2 Anesthesiology/Dentistry and Oral Surgery, Imakiire General Hospital, Kagoshima, JPN

**Keywords:** inflammation, minimally invasive, ultrasound-guided trigeminal nerve block, pulpitis, infection

## Abstract

In aging humans, tooth loss is a predictor of decreased longevity. Tooth loss is mainly caused by dental caries and periodontal disease. Pulpitis refers to inflammation of the dental pulp caused by bacterial infection secondary to dental caries. It is accompanied by severe toothache and has infectious disease-associated pathophysiology. Pulpitis is mainly treated by pulpectomy, which is aimed at removing the infected dental pulp and controlling pain by removing nociceptive nerve fibers. However, teeth without dental pulp have a poor prognosis. In this report, we proposed a novel “super minimally invasive pulp” therapy for treating pulpitis without pulpectomy, which combines antibiotics, steroids, and ultrasound-guided trigeminal nerve block (UGTNB) to protect the dental pulp. UGTNB is used as an analgesic for severe pain, antibiotics for pulp infections, and steroids as antiinflammatory drugs. This novel therapy could improve the longevity of the tooth and thereby oral health.

## Introduction

Oral well-being and functions are important for health and quality of life [1]. The teeth play an important role in mastication, speech, and aesthetics. Tooth loss leads to the loss of these functions and complications, such as nutritional and articulation disorders [2,3]. These conditions may cause depression and social isolation owing to the decreased quality of life [2]. The importance of tooth loss is being increasingly recognized, especially among aging populations. Tooth loss is mainly caused by dental caries and periodontal diseases [3]. According to the 2016 Global Burden of Disease Study, more than 2.44 billion people worldwide suffer from dental caries [4]. In cases with advanced dental caries, the dental pulp undergoes necrosis. The prognosis differs greatly between “vital teeth” (wherein the pulp is still alive) and “nonvital teeth” (wherein the pulp is dead) [5,6]. Suzuki et al. reported that the number of nonvital teeth was associated with tooth loss [6]. They suggested that patients with several nonvital teeth were particularly at risk of tooth loss due to root fractures or caries.

Pulpitis is defined as inflammation of the dental pulp tissue that is mainly caused by bacterial infection secondary to dental caries. Pulpitis can be diagnostically classified as reversible or irreversible. Irreversible pulpitis requires pulpectomy even when the pulp is partially alive. Pulpectomy is an invasive treatment as the blood vessels and nerves that maintain tooth homeostasis are surgically removed. Teeth with their dental pulps removed are classified as nonvital teeth.

The unique inflammatory mechanism of pulpitis can be explained by the fact that the pulp is surrounded by hard tissues such as the enamel and dentin. Pulpitis increases intrapulpal pressure and involves the release of inflammatory substances in response to bacterial invasion, leading to severe tooth pain [7]. Severe pain further exacerbates inflammation by causing the release of inflammatory substances. Hence, symptoms of pulpitis are exacerbated by interactions between infective and inflammatory processes. Pulpectomy aims to eliminate pulpitis and pain sensation by removing the entire pulp tissue including all nociceptive nerve fibers. It was introduced in the 1830s and is a well-established technique for the treatment of severe tooth pain. Most dentists tend to choose pulpectomy for treating pulpitis because of the difficulty in accurately diagnosing reversible or irreversible pulpitis and because they prioritize rapid alleviation of symptoms [8,9]. However, even in cases of irreversible pulpitis, most of the pulp tissue may often be uninfected and uninflamed [8,10].

After pulpectomy, nonvital teeth are associated with some problems. Ricucci et al. reported that bacteria were visualized in most teeth that underwent root canal treatment including pulpectomy [11]. Nixdorf et al. reported that the frequency of all-cause persistent tooth pain after these procedures was estimated to be 5.3%, with higher quality studies suggesting a frequency >7% [12]. Since a tooth is severely damaged by pulpectomy, several prosthetic procedures are required to restore its function. Standard requirements for diagnosis and treatment of pulpitis in recent years include highly precise medical equipment, such as an expensive microscope. Such dental care is available only to a small population, who can afford the costs. In addition, pulpectomy involves many treatment steps and inconveniences for patients. Nonvital teeth have a high risk of root fracture and tooth extraction [6]. Most dentists are aware of the poor long-term prognosis of teeth following pulpectomy. Moreover, prosthetic treatment after tooth loss is not superior to natural teeth in terms of function, regardless of the quality of the oral implant material. Thus, the vitality of the teeth is important for maintaining lifelong oral functions. Therefore, we devised a method to preserve the pulp using nerve blocks and antimicrobial agents in patients with irreversible pulpitis, who are traditionally recommended pulpectomy.

## Technical report

**Figure 1:**
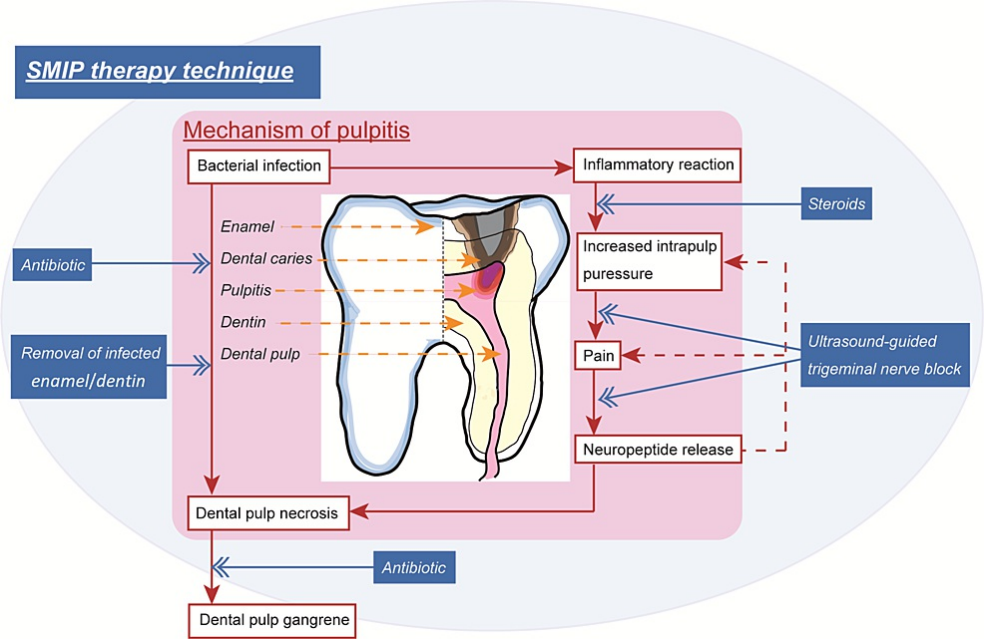
Illustration of the mechanisms mediating pulpitis and the proposed super minimally invasive pulp (SMIP) therapy technique. This depiction includes an illustration of pulpitis caused by caries and information on the causative bacteria. Infection of the dental pulp typically leads to inflammatory reactions, severe pain, and neuropeptide release. Steroids control inflammation, and the ultrasound-guided trigeminal nerve block prevents neuropeptide release by providing analgesia. Moreover, antibiotics and the removal of infected enamel and dentin are effective in arresting dental-pulp necrosis. The figure is created by the authors of this study.

The proposed treatment for pulpitis includes ultrasound-guided trigeminal nerve block (UGTNB) as an analgesic for severe pain, antibiotics for pulp infection, and steroids as antiinflammatory drugs (Figure 1). We have designated this method the “super minimally invasive pulp” (SMIP) therapy and describe it below.

After intravenous administration of antibiotics, UGTNB is performed. For maxillary teeth with pulpitis, ultrasound-guided maxillary nerve block is performed, and for mandibular teeth, ultrasound-guided inferior alveolar nerve block is performed. For local anesthesia, 6 mL of 0.375% ropivacaine is administered after confirming the absence of abnormalities. After confirming sufficient analgesia, a rubber dam is applied. Next, steroids are administered intravenously, and all the infected enamel and dentin are removed. After hemostasis and disinfection of the surgical field, pulp capping is performed with mineral trioxide aggregate. Finally, composite resin or glass ionomer cement is used to restore the tooth.

## Discussion

Ultrasound-guided nerve blocks have been effectively used peri- or post-operatively. This analgesic method has many benefits compared with nonsteroidal antiinflammatory drugs and opioids [13,14]. UGTNB is applied in patients with maxillofacial fractures or those undergoing oral cancer surgery to exert a strong analgesic effect for approximately 24-72 h [14]. Since UGTNB does not require general anesthesia or sedation, it is appropriate for providing analgesia in pain clinics [15].

Porporatti et al. reported that patients with pulpitis experienced neuropathic pain and somatosensory alterations not only before treatment but also after pulpectomy [16]. Various theories regarding the cause of this phenomenon have been proposed, and importantly, a relationship between the parabrachial nucleus and trigeminal nerve has recently been reported [17]. Nociceptive information in the trigeminal nerve area is synaptically mediated in the parabrachial nucleus and is then projected to the limbic nuclei involved in pain and emotions. Thus, pain in the trigeminal nerve-innervated area and facial pain might be easily influenced by emotions [17]. Residual pain after pulpectomy and prolonged pain can increase patients' anxiety and fear and induce chronic orofacial pain. Therefore, UGTNB, which provides prolonged and powerful analgesia may be useful in patients with pulpitis.

Antibiotics are usually prescribed in dentistry for pericoronitis and osteomyelitis of the jaw. However, their use in pulpitis is debated. In many cases, pulpectomy is sufficient to alleviate the symptoms of pulpitis, and antibiotics are ineffective after pulpectomy because the blood flow is lost. If the treatment strategy is to preserve the pulp, the use of antibiotics might be beneficial because blood vessels are preserved even in the infected pulp. Causative bacteria for dental caries have been identified and are well-known. Therefore, choosing the antimicrobial agent is not difficult, and the minimum required dose of antibiotics can be administered. Histological studies regarding the extent of bacterial invasion in the initial stage of pulpitis have shown that the infected portion of the pulp comprises a very small area [8,10]. Hence, antibiotic administration and removal of the infected dentin or enamel are usually adequate to control the infection.

Steroids are antiinflammatory medicines used to treat a range of conditions, including asthma and chronic obstructive pulmonary disease, painful joints or muscles, inflammatory bowel disease, and multiple sclerosis. Prolonged use of steroids causes atrophy of the adrenal glands, which reduces their ability to produce steroids. However, short-term or low-dose steroid therapy tends to cause no significant side effects. Careful evaluation of the patient's condition is required prior to using steroids.

SMIP therapy is significantly different from conventional dental pulp therapy in that there is no surgical invasion of the dental pulp regardless of its condition. It is important to remove all infected enamel and dentin, regardless of the amount of dental pulp exposed. Preserving the dental pulp directly contributes to the improvement of tooth prognosis. In case of secondary caries, the presence of the dental pulp could help prevent the spread of infection. Nonvital teeth tend to be asymptomatic until the caries infection progresses to apical periodontitis. This increases the risk of tooth loss. Even a small area of healthy dental pulp may prevent secondary infection from caries because of the preserved immune system and blood flow. Further, the limitations of root canal therapy after removing the dental pulp must be considered. Insufficient sealing of the root canal could result in microleakage and lead to apical periodontitis in the future [18]. In many cases, tooth tissue removal increases the risk of microleakage and subsequent reinfection. Pulpitis may progress to dental pulp necrosis in the absence of appropriate treatment. However, it is unclear if it is better to retain or remove partially necrotic tissue. Diseases that cause necrosis include myocardial and cerebral infarctions, in which it is both unnecessary and harmful to remove the necrotic tissue because the disadvantages of surgical removal greatly outweigh the advantages. We believe that partially necrotic pulp tissue should be followed up without removal because preservation of as much tooth tissue as possible is important.

Research on novel approaches and pulp-regeneration treatments while protecting the pulp is progressing, and the importance of the pulp is being reaffirmed [19]. Further, inexpensive and portable ultrasound devices have lowered the cost of ultrasound-guided nerve blocks [20].

## Conclusions

Pulpectomy is commonly performed in the treatment of pulpitis to improve symptoms. However, there is a dilemma when choosing this procedure as the prognosis of the tooth is poor after pulpectomy. We described a novel treatment for pulpitis, termed “SMIP” therapy. We hypothesize that this new therapeutic approach can be adopted in teeth with pulpitis to improve tooth longevity and patients’ quality of life with minimal invasion of the pulp. Further studies are required to examine procedural details, safety, success rates, and efficacy of the SMIP therapy.
